# Effective Treatment of Respiratory Alphaherpesvirus Infection Using RNA Interference

**DOI:** 10.1371/journal.pone.0004118

**Published:** 2009-01-05

**Authors:** Amy Fulton, Sarah T. Peters, Gillian A. Perkins, Keith W. Jarosinski, Armando Damiani, Margaret Brosnahan, Elizabeth L. Buckles, Nikolaus Osterrieder, Gerlinde R. Van de Walle

**Affiliations:** 1 Department of Microbiology and Immunology, College of Veterinary Medicine, Cornell University, Ithaca, New York, United States of America; 2 Department of Clinical Sciences, College of Veterinary Medicine, Cornell University, Ithaca, New York, United States of America; 3 Department of Biomedical Sciences, College of Veterinary Medicine, Cornell University, Ithaca, New York, United States of America; 4 Institut für Virologie, Freie Universität Berlin, Berlin, Germany; Cambridge University, United Kingdom

## Abstract

**Background:**

Equine herpesvirus type 1 (EHV-1), a member of the *Alphaherpesvirinae*, is spread via nasal secretions and causes respiratory disease, neurological disorders and abortions. The virus is a significant equine pathogen, but current EHV-1 vaccines are only partially protective and effective metaphylactic and therapeutic agents are not available. Small interfering RNAs (siRNA's), delivered intranasally, could prove a valuable alternative for infection control. siRNA's against two essential EHV-1 genes, encoding the viral helicase (Ori) and glycoprotein B, were evaluated for their potential to decrease EHV-1 infection in a mouse model.

**Methodology/Principal Fndings:**

siRNA therapy *in vitro* significantly reduced virus production and plaque size. Viral titers were reduced 80-fold with 37.5 pmol of a single siRNA or with as little as 6.25 pmol of each siRNA when used in combination. siRNA therapy *in vivo* significantly reduced viral replication and clinical signs. Intranasal treatment did not require a transport vehicle and proved effective when given up to 12 h before or after infection.

**Conclusions/Significance:**

siRNA treatment has potential for both prevention and early treatment of EHV-1 infections.

## Introduction

Equine herpesvirus type 1 (EHV-1) is a major cause of respiratory, neurologic and reproductive disease in horses worldwide. EHV-1 is a member of the *Alphaherpesvirinae* and closely related to the causative agents of human chicken pox/shingles (varicella zoster virus, VZV) as well as cold sores and genital herpes, herpes simplex types 1 (HSV-1) and 2 (HSV-2) [1]. EHV-1 is spread through respiratory secretions and replicates in the nasal epithelium upon gaining access to the respiratory tract. Initial replication is followed by a leukocyte-associated viremia. Further replication of EHV-1 can occur in endothelia of blood vessels of the central nervous system (CNS) and the uterus, where vasculitis can lead to myeloencephalopathy or abortion, respectively [2]. EHV-1 establishes latency in neural and lymphoid tissue and recrudesces in times of stress such as transportation, pregnancy, competitions, and racing [3]. Therefore, horses traveling to competitions and coming into contact with new animals are at risk of shedding or contracting the virus, making this viral infection a huge concern for the performance horse industry. Recently, an increased occurrence of neurologic EHV-1 outbreaks, especially of the neurologic form of the disease, has been reported, and these outbreaks are more and more frequently associated with high mortality rates. Consequently, EHV-1 has recently been classified as a potentially emerging disease by the US Department of Agriculture [4].

Unfortunately, vaccination is currently the only form of EHV-1 control and the available vaccines do not provide complete protection. As EHV-1-induced protective immunity is only short-lived, vaccination has to be repeated at least every 6 months [5]. However, the continuing EHV-1 outbreaks involving large numbers of animals raises the question as to whether repeated vaccination by itself is sufficient to protect animals in outbreak situations. Moreover, repeated vaccination has recently been suggested to predispose horses to develop the most severe form of EHV-1 infection, myeloencephalopathy [5]–[7]. Treatment of EHV-1 infections is reduced to symptomatic care and experimental antiviral drugs have yet to prove clinical efficacy. For example, drugs such as acyclovir or valacyclovir were shown to have serious limitations due to poor bioavailability and high therapeutic concentrations are needed in order to reach the desired effect, making these drugs a costly medication with questionable efficacy [8]–[10].

RNA interference mediated by small interfering RNA's (siRNA's) is an important defense mechanism against viral infections in plants. siRNA's have recently been intensively examined for their ability to prevent and/or treat viral infections. siRNA's bind to complementary target mRNA and, upon interaction with the cellular RNA interference mechanisms, will specifically target these sequences for degradation, resulting in inhibition of protein expression [11], [12]. Synthetic siRNA's have been shown to successfully inhibit viral replication of several viruses, among them herpesviruses [13]–[15]. In addition, siRNA's against respiratory viruses have been tested extensively in animal models. It has been demonstrated that siRNA's effectively inhibited the replication of SARS coronavirus in rhesus macaques [16]. Additionally, intranasally administered siRNA's targeting viral genes of parainfluenza virus and respiratory syncytial virus were shown to be effective in preventing pulmonary disease and pathology in mice [17].

In the present study, we evaluated the use of siRNA therapy to limit and prevent EHV-1 infection *in vitro* and in a murine model of EHV-1 infection. Synthetic siRNA's were designed against two essential and highly conserved EHV-1 genes. Firstly, the open reading frame (ORF) 33 encoding the envelope glycoprotein B (gB) was chosen because this glycoprotein is essential for viral entry and cell-to-cell spread [18]. Secondly, siRNA's against ORF53, encoding the origin-binding protein (Ori) helicase, were designed as this protein is essential for the initiation of herpesviral genome replication [19], [20].

## Results

### Treatment with siRNA targeting glycoprotein B or the origin-binding protein helicase is effective in reducing EHV-1 replication *in vitro*

**Figure 1:**
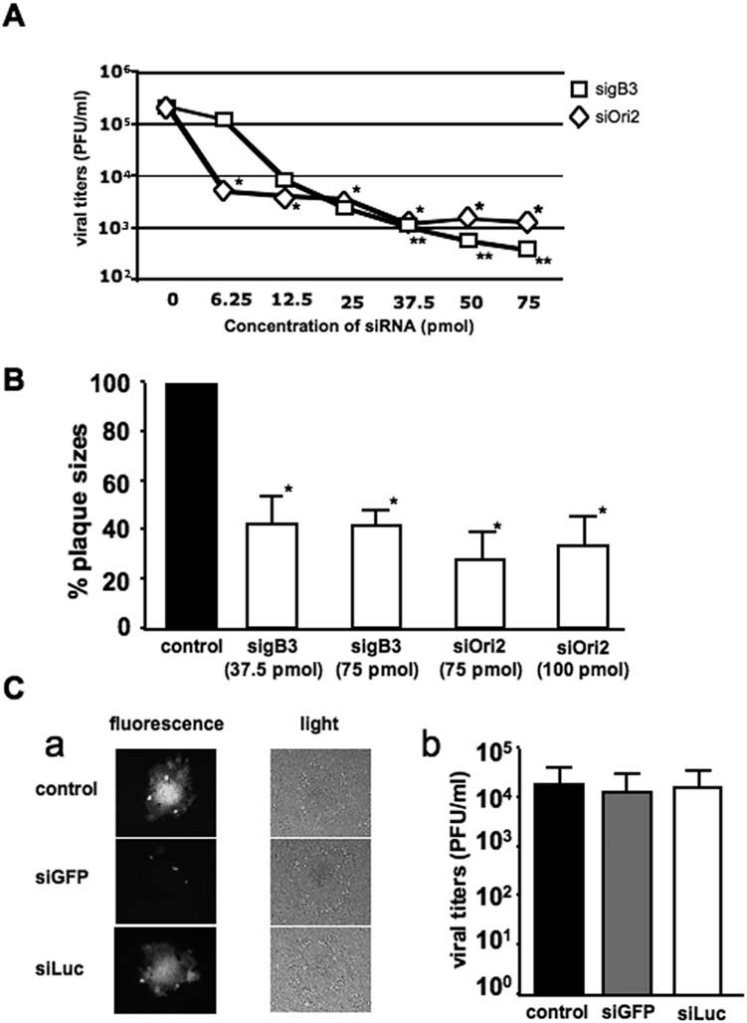
Effect of siRNA's targeting glycoprotein B (gB) and origin-binding protein (ori) helicase on EHV-1 replication and cell-to-cell spread. (A). siRNA's targeting gB (sigB3) or Ori (siOri2) were transfected into RK13 cells and cells were infected with 100 PFU of EHV-1 strain rAb4Δgp2 14 h later. Supernatants were collected 24 h p.i. from sigB3- (□) and siOri2-transfected cells (◊), and viral titers were determined with standard plaque assays. Asterisks indicate statistically significant differences (*: p<0.05, **: p<0.01). (B). Cells were fixed with 10% formalin and the average plaque areas were determined. Asterisks indicate statistically significant differences (p<0.05). (C). As controls cells were included, which were either not transfected with siRNA or were transfected with 75 pmol siGFP or siLuc before infection with rAb4Δgp2. Representative pictures were taken with light and fluorescent microscopy (a) and viral titers were determined with standard plaque assays (b).

EHV-1 glycoprotein B (gB), an envelope protein essential for viral entry into cells and cell-to-cell spread [18], and origin-binding protein (Ori) helicase, an enzyme necessary for EHV-1 genome replication [19], were chosen as possible targets for RNA interference. siRNA's against these proteins were synthesized and evaluated for their effectiveness to reduce EHV-1 replication. sigB3, directed against gB mRNA, efficiently reduced viral replication in a dose-dependent manner. A 10-fold reduction of viral titers was observed with as low as 6.25 pmol sigB3 (p<0.05) and reached a steady-state level starting at 37.5 pmol with up to a 100-fold reduction in viral titers (p<0.01) (Figure 1A). Treating cells with siOri2, directed against the Ori helicase mRNA, resulted in a 50-fold reduction in viral titers, at a concentration ranging between 37.5 and 75 pmol (p<0.05). Furthermore, these siRNA's were also able to significantly reduce plaque sizes, with up to a 60% reduction in total plaque area when 37.5 pmol sigB3 or 75pmol siOri2 was used (p<0.05) (Figure 1B).

To evaluate siRNA transfection efficiency, cells were transfected with commercially available siGFP, an siRNA targeting the *egfp* gene. eGFP-expressing EHV-1 strain rAb4Δgp2 was used for *in vitro* studies because eGFP expression allows for ready identification of infected cells and virus-induced plaques. Pre-treating cells with 75 pmol siGFP efficiently reduced eGFP expression in infected cells (Figure 1C), without having any effect on EHV-1 infectivity, i.e. plaque sizes and titers remained unaffected even though eGFP expression was no longer detectable (Figure 1C). To ensure that siRNA transfection by itself did not have a negative effect on EHV-1 infectivity, cells were treated with 75 pmol of the negative control siRNA siLuc, an siRNA targeting the luciferase gene, before infection. As expected, because the rAb4Δgp2 does not express luciferase, we did not observe any effect on eGFP expression (Figure 1C) or on viral infectivity when compared to mock-treated cells (Figure 1C).

**Figure 2:**
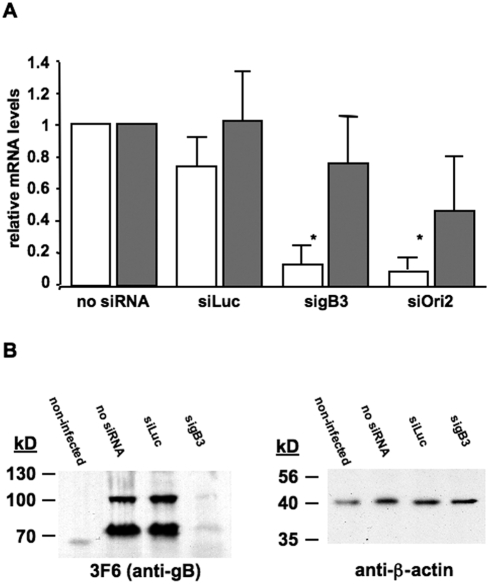
siRNA's efficiently suppresses gB and Ori expression at the mRNA and protein level. (A). Relative qRT-PCR. RK13 cells were transfected with 37.5 pmol sigB3, 75 pmol siOri2, 75 pmol siLuc or not transfected. Cells were infected 14 h later with 500 PFU of rAb4Δgp2 and at 12 h p.i., RNA was extracted from infected cells. RT-PCR was used to determine relative levels of gB or Ori (white bars) or EHV-1 IR6 (grey bars) mRNA, using rabbit β-actin as the endogenous housekeeping gene. Asterisks indicate statistically significant differences (p<0.05). (B). Western blot analysis. RK13 cells were either not transfected or transfected with 75 pmol of the control siRNA siLuc or 75 pmol sigB3. Cells were infected 14 h later with 500 PFU of rAb4Δgp2. At 24 h p.i., cell lysates were prepared and analyzed by SDS-PAGE under reducing conditions. Anti-gB mAb 3F6 (1/500) and anti-β-actin (1/5000), followed by anti-mouse IgG peroxidase (1/7500) were used. Cell lysates from non-infected RK13 cells were included as a control.

To assess the effectiveness of sigB3 and siOri2 at the mRNA level, relative quantitation of the two mRNA's was performed using quantitative reverse transcriptase PCR (qRT-PCR). At 12 h p.i., the relative quantity of gB or Ori mRNA was reduced by more than 90% following sigB3 or Ori2 treatment, respectively, when compared to untreated cells (p<0.05) (Figure 2A). No significant difference in gB or Ori mRNA levels in cells was observed between (i) untreated cells and cells treated with the control siRNA siLuc (Figure 2A), and (ii) the control siRNA's siGFP and siLuc (data not shown). The effectiveness of silencing gB was also evaluated at the protein level by western blot analysis. Using the anti-gB antibody 3F6, a clear reduction in protein expression was observed in cells treated with sigB3 compared to non-treated cells or cells treated with control siLuc siRNA (Figure 2B). Mock-infected cells did not express gB and the control antibody against β-actin showed that equal amounts of cell proteins were loaded in each lane (Figure 2B).

We concluded from the data that siRNA treatment targeting EHV-1 gB or Ori can significantly reduce EHV-1 infection by effectively silencing gB or Ori expression at the mRNA and protein level. The silencing of these two essential EHV-1 proteins resulted in significantly reduced viral titers and plaque sizes.

### Treatment with siRNA targeting two essential EHV-1 genes has an additive effect on reduction of EHV-1 replication *in vitro*

**Figure 3:**
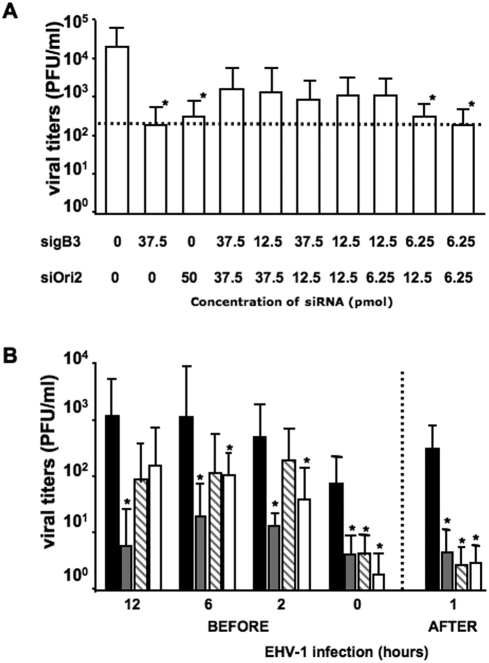
Combining siRNA's have an additive effect on reduction of EHV-1 replication and are effective before and after infection. (A). The siRNA's sigB3 and siOri2 were transfected into RK13 cells, either alone or in different combinations, and cells were infected 14 h later with 500 PFU of rAb4Δgp2. Supernatants were collected 24 h p.i. and viral titers were determined with standard plaque assays. Asterisks indicate statistically significant differences (p<0.05). (B). RK13 cells were transfected with 75 pmol control siRNA (black bars), 37.5 pmol sigB3 (grey bars), 75 pmol siOri2 (hatched bars) or a combination of 6.25 pmol sigB3 and 6.25 pmol siOri2 (white bars). At different times after transfection (ranging form 12 h up to 0 h) cells were infected with 500 PFU of rAb4Δgp2 and supernatants were collected at 24 h p.i. In one set of experiments, cells were first infected with rAb4Δgp2 and transfected with the different siRNA's 1 h later. Viral titers were determined with standard plaque assays. Asterisks indicate statistically significant differences (p<0.05).

In the next series of experiments we repeated treatment but used combinations of the two siRNA's. When applied together, gB3 and siOri2 showed the same effectiveness in reducing EHV-1 infectivity, but at a much lower concentration than either siRNA by itself. As seen in Figure 3A, the 80-fold reduction in viral titers observed after treatment with either 37.5 pmol sigB3 or 50 pmol siOri2 was also obtained when using a combination of 6.25 pmol sigB3 and 6.25 pmol siOri2 (p<0.05). Surprisingly, a combination of higher concentrations of sigB3 and siOri2 (e.g. 37.5 pmol each or 12.5 pmol each) was less effective in reducing viral titers than the lower concentration of 6.25 pmol each (Figure 3A). Such an observation has also been reported for siRNA combinations against human respiratory viruses, where the inhibitory activity of one siRNA was inhibited when using high amounts of another siRNA at the same time [17]. It is known that cells have a limited capacity to assemble the RNA-induced silencing complex (RISC) onto transfected siRNA and that siRNA's differ in their specificity and efficiency of RISC targeting [21]. A possible explanation could therefore be that both siRNA's compete for the available RISC pool present in the cell and as such inhibit each other at higher concentrations. The effectiveness of this 6.25 pmol combination cocktail on gB silencing was also evaluated with both qRT-PCR and Western blot analysis and resulted in similar observations as described before with 37.5 pmol sigB3 (data not shown). These data indicate that targeting multiple genes simultaneously is as effective in reducing viral replication as when one gene is targeted, and does so with significantly lower concentrations of each of the individual siRNA's.

### sigB3 and siOri2, alone or in combination, also inhibit viral replication after infection

Finally, the last set of *in vitro* experiments evaluated the efficacy of siRNA addition in function of time. Cells were transfected with siRNA's at different time points before (12, 6 and 2 h), simultaneously, and 1 h after infection. It was observed that sigB3 (37.5 pmol) could significantly reduce viral replication when applied at any time between 12 h prior to and up to 1 h after infection (p<0.05) (Figure 3B). siOri2 at a concentration of 75 pmol reduced viral titers significantly when transfection and infection occurred simultaneously or when siOri2 was transfected 1 h after infection (p<0.05) (Figure 3B). The combination of sigB3 and siOri2 at 6.25 pmol each, significantly decreased viral titers at all time points tested (p<0.05), with the exception of 12 h before infection (Figure 3B). There was no significant difference at all time points tested between the control treatments that included cells treated with siGFP, siLuc or no siRNA (data not shown). Taken together, the *in vitro* experiments clearly showed that siRNA treatment is not only effective in reducing EHV-1 replication when used before infection, but is as efficient, if not even more, when applied during or after the onset of EHV-1 replication.

### Intranasal administration of siRNA reduces clinical sings and viral replication in a murine model of EHV-1 infection

The efficiency of siRNA therapy *in vitro* clearly showed that cellular replication and spread of EHV-1 can be prevented in cell culture. A murine model was used to assess whether siRNA treatment is also effective *in vivo*. This mouse model of EHV-1 infection, first described by Awan et al. in 1990 [22], has been intensively used to predict the efficacy of putative EHV-1 vaccines in horses [23]–[26], as well as the virulence potential of several EHV-1 strains [27]–[32]. In the present study, mice were anaesthetized and inoculated intranasally with siRNA's, followed by infection with 1×10^5^ PFU Ab4 by the same route. The amounts and combinations of siRNA's used, as well as the time points of siRNA treatment and EHV-1 infection are summarized in Table 1.

**Table 1:** Protocol outline for the siRNA transfection/infection experiments in mice.

group	concentration/combination of siRNA's (pmol)	time of siRNA application	Transfection reagent	Infection
A	sigB3 (187.5)	24 h before I^a^	lipofectamine	Ab4
B	sigB3 (187.5)	0.5 h before I	lipofectamine	Ab4
C	siOri2 (187.5)	24 h before I	lipofectamine	Ab4
D	siOri2 (187.5)	0.5 h before I	lipofectamine	Ab4
E	sigB3/siOri2 (31.25/31.25)	24 h before I	lipofectamine	Ab4
F	sigB3/siOri2 (31.25/31.25)	0.5 h before I	lipofectamine	Ab4
G	sigB3/siOri2 (62.5/62.5)	24 h before I	lipofectamine	Ab4
H	sigB3/siOri2 (62.5/62.5)	0.5 h before I	lipofectamine	Ab4
I	siLuc (250)	24 h before I	lipofectamine	Ab4
J	no siRNA	24 h before I	lipofectamine	Ab4
K	siLuc (250)	12 h before I	lipofectamine	Ab4
L	siLuc (250)	12 h before I	PBS	Ab4
M	sigB3/siOri2 (62.5/62.5)	12 h before I	lipofectamine	Ab4
N	sigB3/siOri2 (62.5/62.5)	6 h before I	lipofectamine	Ab4
O	sigB3/siOri2 (62.5/62.5)	12 h before I	PBS	Ab4
P	sigB3/siOri2 (62.5/62.5)	6 h before I	PBS	Ab4
Q	sigB3/siOri2 (62.5/62.5)	1 h after I	PBS	Ab4
R	sigB3/siOri2 (62.5/62.5)	6 h after I	PBS	Ab4
S	sigB3/siOri2 (62.5/62.5)	12 h after I	PBS	Ab4
T	sigB3/siOri2 (62.5/62.5)	24 h after I	PBS	Ab4
*control* b	*no siRNA (PBS)*	*24 h before*	*medium*	*medium*
*control*	*no siRNA (PBS)*	*12 h before*	*medium*	*Medium*

a I: infection.

b *control*: to evaluate weight loss due to anesthesia.

**Figure 4:**
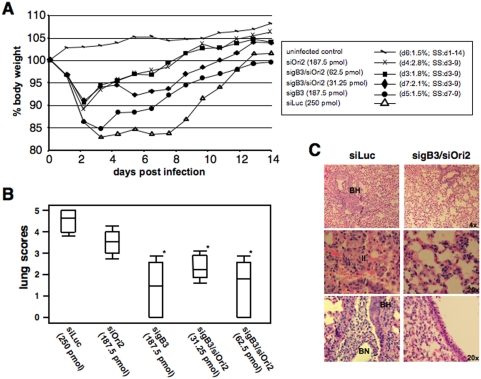
siRNA's are effective in reducing inflammatory responses in a murine model of EHV-1 infection when applied 0.5 h before infection. (A). Development of mean body weights after infection. Balb/c mice (groups of 12) were transfected intranasally with sigB3 and siOri2, alone or in combination, and mice were infected intranasally with 1×10^5^ PFU of Ab4 0.5 h later. Mice inoculated with 75 pmol siLuc were used as positive controls and uninfected negative control mice were also included. Mean body weights were determined on the day of infection (day 0) up to day 14 p.i. Mean body weights on the day of infection were set to 100%. Standard deviations (SD's) ranged from 0.7 to 2.8%. The day of the maximal SD's for each group are indicated in brackets. The days where statistical significant differences (SS) were observed, as determined with non-parametric Wicoxon-Whitney and Kruskal Wallis analyses, are also given between brackets. Histopathology. A total lung score was determined at day 2 p.i. in three mice of each group and graded on a scale of 0+ (normal) to 5+ (severe), as described in Materials and Methods. Asterisks indicate statistically significant differences (p<0.05) (B). Representative H&E images showing histological features in lungs of mice treated with the control siLuc and mice treated with siRNA's against EHV-1 genes. BH: bronchiolar hyperplasia, II: interstitial inflammation, BN: bronchiolar necrosis (C).

In an initial experimental set up, siRNA treatment with sigB3 and siOri2, given alone or in combination at 24 h and 0.5 h before infection with EHV-1, was evaluated. Mice (12 per group) treated with the control siRNA siLuc began losing weight as early as 1 day p.i. and continued to lose weight until day 3 p.i., when a maximum weight loss of up to 17% of their original body weights was observed. Pre-infection weights were regained only at day 12 p.i. (Figure 4A). These results were comparable to those obtained from mice that only received the siRNA transfection reagent lipofectamine before infection (data not shown). Mice treated with siRNA's targeting EHV-1 genes 0.5 h before infection also lost weight during the first 2 days p.i., but this weight loss only reached a maximum of 10% for the mice treated with 187.5 pmol siOri2 and the combination sigB3/siOri2 (31.25 pmol and 62.5 pmol, respectively). Mice treated with 187.5 pmol siOri2 and 62.5 pmol sigB3/siOri2 regained their original body weight by day 8 p.i., and mice treated with 31.25 pmol sigB3/siOri2 by day 10 p.i. (Figure 4A). The group treated with 187.5 pmol sigB3 lost up to 15% of their body weight and took until day 14 p.i. to regain their pre-infection weights. However, mice started regaining weight already by day 3 p.i., which is in contrast to the control siLuc treated group where the mice didn't start regaining weight until day 8 p.i. The differences between sigB3/siOri2-treated mice and those treated with control siRNA's or transfection reagents alone were statistically significant on days 3–9 p.i. (non-parametric statistical testing, p<0.05) (Figure 4A). The uninfected control group did not show any weight loss, indicating that the repeated anesthesia protocols used for the intranasal application of siRNA's and virus did not have any effect on weight loss (Figure 4A). Since body weight loss caused by EHV-1 infection is strongly associated with an inflammatory response in lungs of infected mice, we validated the data on weight loss by histopathological analyses of lung tissues obtained on days 2 and 4 p.i. The lungs of mice treated with control siLuc had the most pronounced hyperplasia of bronchiolar epithelium at day 2 p.i., the most necrotic cells in the airway lumina, the most inclusion bodies as well as the most significant interstitial inflammation (Figure 4B&C). Lungs of mice treated with sigB3 were slightly less affected and lungs of mice treated with siOri2 or the sigB3/Ori2 combinations were clearly less affected compared to the siLuc control group (Figure 4B&C).

**Figure 5:**
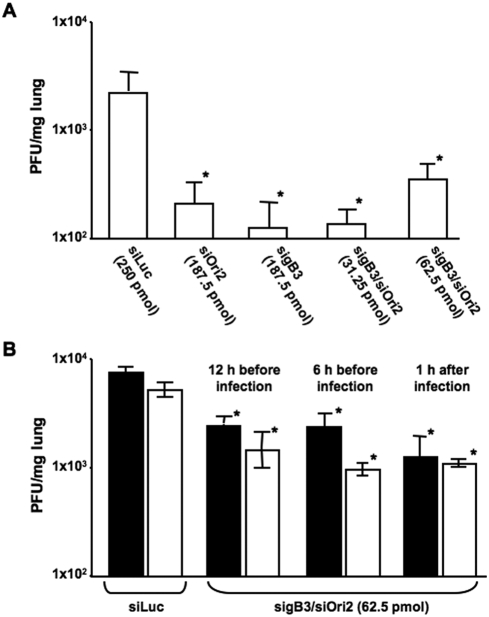
siRNA's are effective in reducing viral replication when applied before infection, even in the absence of a transfection reagent. In a first experiment, Balb/c mice (groups of 12) were transfected intranasally with sigB3 and siOri2, alone or in combination, and mice were infected intranasally with 1×10^5^ PFU of Ab4 0.5 h later (A). In a second experiment, Balb/c mice (groups of 12) were inoculated intranasally with 62.5 pmol sigB3/siOri2, complexed with lipofectamine (closed symbols) or in PBS (open symbols) 6 or 12 h before infection with 1×10^5^ PFU of Ab4 (B). Mice transfected with 75 pmol siLuc were used as positive controls and viral titers were determined in three mice of each group on day 2 p.i. Titers in lungs and standard deviations are shown. Asterisks indicate statistically significant differences (p<0.05) between mice transfected with sigB3 and siOri2, alone or in combination, and mice transfected with the control siRNA siLuc.

With regard to virus titers, mice inoculated with control siLuc had titers around 3×10^3^ PFU/mg lung tissue on day 2 p.i. (Figure 5A). In contrast, virus titers in lungs of mice treated 0.5 h before infection with sigB3 and siOri2, either alone or in various combinations, showed a significant reduction in viral titers with an average of 3×10^2^ PFU/mg lung tissue (Student's t-test, p<0.05) (Figure 5A). A similar reduction in virus titers was observed on day 4 p.i., although declining virus titers at that day generally indicates beginning clearance of the virus infection (data not shown). Treatment with siRNA's targeting EHV-1 genes at 24 h before infection also showed a reduction in body weight loss and virus titers to some extent when compared to the control siRNA group, albeit without reaching statistical significance. All infected mice showed maximal weight loss at day 4 p.i., with around 20% in the group of mice that received the control siLuc and only 15–17% in mice treated with siRNA's against EHV-1 genes. In addition, original body weights were regained 1 or 2 days earlier in the treated groups, compared to mice treated with control siLuc, in which pre-infection weights were only reached as late as 13 days p.i. Titers of 1.7±7.7×10^3^ PFU/mg lung tissue were observed in the siLuc-treated group, which were slightly higher than in lungs of mice treated with sigB3 (4.4±4.2×10^2^ PFU/mg), siOri2 (1.4±6.5×10^3^ PFU/mg) or the sigB3/siOri2 combinations (1.6±1.3×10^2^ PFU/mg and 1.3±1.0×10^3^ PFU/mg for 31.25 pmol sigB3/siOri2 and 62.5 pmol sigB3/siOri2 respectively). Taken together, this first *in vivo* experiment clearly revealed that siRNA therapy is effective *in vivo* by reducing clinical symptoms in challenge-infected animals and was capable of significantly reducing inflammation and viral replication in the target organ, the lung, at least when applied 0.5 h before infection.

Since it has been described previously that siRNA application to mucosal surfaces *in vivo* does not require a transfection vehicle [17], a second experiment in mice was performed without a transfection agent. In addition, to extrapolate our previous findings to other time points, we included siRNA treatments 12 h and 6 h before infection with EHV-1. The siRNA combination of 62.5 pmol sigB3/siOri2 was evaluated, since this treatment group was shown to significantly reduce both weight loss and virus replication. First, it was observed that intranasal application of sigB3/siOri2 did not require a transfection reagent because no significant differences in weight loss (data not shown) and viral titers (Student's t-test, p<0.05) (Figure 5B) were observed when siRNA's were delivered with either lipofectamine or PBS. Secondly, it was observed that siRNA treatment 12 h before infection was effective in significantly reducing both weight loss (non-parametric statistical testing, p<0.05, data not shown) and virus replication in the lungs (Student's t-test, p<0.05) (Figure 5B). Treatment with 62.5 pmol sigB3/siOri2 6 h before EHV-1 infection did not significantly reduce weight loss (data not shown); however, a significant reduction of virus titers in the lungs 2 days p.i. was observed in these mice (Student's t-test, p<0.05) (Figure 5B).

**Figure 6:**
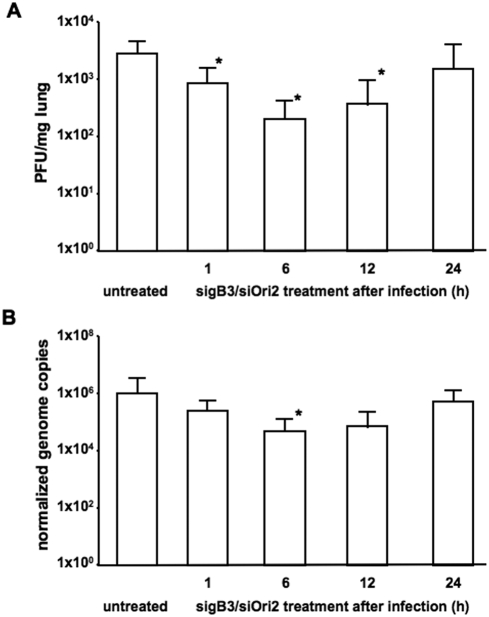
siRNA's are effective in reducing viral replication when applied after infection. Balb/c mice (groups of 15) were infected intranasally with 1×10^5^ PFU of Ab4. At 1, 6, 12 or 24 h post-infection, mice were inoculated intranasally with 62.5 pmol sigB3/siOri2, in PBS. (A). Viral titers were determined in lungs of five mice per group on day 3 p.i. by co-cultivation. Titers in lungs and standard deviations are shown. (B). Viral loads in lung tissues were also measured by qPCR and are plotted as EHV-1 genome (IR6 gene) copies per million mouse iNOS gene copies. Asterisks indicate statistically significant differences (p<0.05; Student's t-test) between untreated mice and mice inoculated with 62.5 pmol sigB3/siOri2.

Lastly, a third experiment was conducted to evaluate the effect of siRNA treatment after infection. Mice (15 per group) were infected with Ab4, followed by siRNA inoculation at 1, 6, 12 and 24 h after infection. The control group consisted of 15 untreated infected mice as we could never observe any significant differences between untreated and siLuc-treated mice in our previous experiments. Viral loads in lungs were determined by viral titrations and qPCR in five mice per group on day 3 p.i. The amount of infectious virus, as determined by virus isolation, in lungs of mice treated with siRNA's at 1, 6 and 12 h infection was significantly reduced (Student's t-test, p<0.05) compared to untreated infected animals (Figure 6A). No significant reduction in virus titers was observed in animals treated with siRNA's 24 h post infection (p = 0.9). A reduction in viral genome copies in the lungs of infected mice was observed at 1, 6 and 12 h p.i., but this reduction only reached statistical significance in the group of mice that were treated with siRNA's 6 h post infection (Student's t-test, p<0.05; Figure 6B). These data show that treatment with siRNA's after infection is also efficient in reducing EHV-1 replication and therefore indicate the potential of siRNA treatment during an EHV-1 outbreak where several individuals might already have been exposed to the virus.

## Discussion

Over the course of the past few years, much progress has been made in evaluating RNA interference as a novel potent therapy in the fight against a number of virus infections of man and animals. This progress has led to serious promise in terms of drug development using small interfering (si)RNA technology and intranasal application. For example, intranasal siRNA application against respiratory syncytial virus (RSV) has recently entered phase I clinical trials and it was shown that siRNA was safe and well tolerated in healthy volunteers [33], [34]. This area of research is filling a therapeutical void and has provided evidence that siRNA can possibly be developed into a treatment and/or prevention option for respiratory viruses that are otherwise difficult to control.

In the present study, we describe the use of siRNA against an important respiratory pathogen in horses, the alphaherpesvirus equine herpesvirus type 1 (EHV-1). EHV-1 is a major pathogen which defies control measures as demonstrated by the increase in severe, and often fatal, disease outbreaks which occur despite vaccination [7]. siRNA's directed against highly conserved essential EHV-1 genes were shown in an *in vitro* setting to significantly reduce viral replication as measured by a significant reduction in both number and size of plaques. The results indicated that the selected siRNA's interfered with EHV-1 infectivity and were capable of decreasing virus spread to neighboring uninfected cells. The application of each siRNA was readily effective, but when combined, a significant reduction in infectivity was already observed when as little as 6.25 pmol of each siRNA per 1×10^6^ cells in 2 mL of culture medium were used. Interestingly, the effective concentration of our siRNA was far below the *in vitro* siRNA concentrations described for other respiratory pathogens like RSV, parainfluenza virus or SARS coronavirus [35], [36], indicating the potency of the selected siRNA's against EHV-1 genes tested in this study.

Using the well-established *in vivo* murine model of EHV-1 infection, we were also able to demonstrate that mice treated intranasally with EHV-1-specific siRNA's were protected against clinical signs like weight loss. Importantly, the viral loads in the lungs of treated mice were significantly lower as assessed by (i) viral titration of lung tissues, (ii) quantitative real-time PCR and (iii) histological evaluation of inclusion bodies. In addition, histological evaluation of infected lung tissues of mice treated with control siRNA's directed against luciferase showed extensive perivascular cuffing as well as interstitial inflammatory inflammation. In contrast, significantly less inflammatory infiltrations and vascular changes were observed in mice treated with siRNA's that specifically target EHV-1 genes. Another promising result of these initial *in vivo* mice studies was that no significant difference could be observed between the effectiveness of siRNA complexed with the transfection reagent lipofectamine or with buffer (PBS). This agrees with previous reports where strong activity of intranasally administered naked siRNA was demonstrated in various animal models [16], [17], [37].

One concern about using RNA interference against viruses is escape from siRNA by mutation of the targeted sequences under siRNA pressure. Mutational changes have been reported for RNA viruses like human immunodeficiency or hepatitis C virus [38], [39], but no such escape mutation was found upon siRNA treatment against other herpesviruses like HSV-2 [15]. Also in the present study, sequence analysis of EHV-1 viral DNA isolated from lungs of siRNA–treated infected mice did not show any mutations in the targeted gB and Ori genes (data not shown), indicating that escape mutation might not be as concerning for DNA viruses. In addition, we used a combination of siRNA's against two different essential genes, hereby further reducing the likelihood of escape mutations.

Another concern raised is the question of whether the effect observed upon siRNA addition is caused by the induction of an immune response, e.g. IFN-γ responses, upon delivery of the nucleic acids or by a specific action of the chosen siRNA. We did not focus extensively on the potential induction of an innate immune response following application of siRNA against EHV-1. However, it has been previously reported that interferon responses mostly occur *in vitro* and depend more on the transfection reagent than the actual siRNA [40], [41]. In addition, several *in vivo* studies, including one assessing intranasally applied siRNA against RSV, were unable to reveal any siRNA-induced interferon responses [17], [42]. These data led us to assume that the induction of an innate immune response upon intranasal administration of siRNA against EHV-1 genes would also be unlikely. Furthermore, the control siRNA's siLuc and siGFP both failed to have any silencing effect on EHV-1, lending additional support for the notion that the observed effects using gB- and Ori-specific siRNA's are indeed specific.

A major bottleneck in the development of siRNA therapy is optimization of the potency and half-life of siRNA's in order to be efficacious in a realistic clinical setting. What modifications are optimal will likely depend on the clinical indication and the strategy used to deliver the siRNA. For example, incorporation into complexes and particles has been shown to offer quite variable protection of siRNA from exposure to endogenous nucleases [43]. Therefore, unmodified siRNA's may be particularly useful in situations in which long-term silencing is not required, such as treating acute viral infections in humans and animals. Notably, many of the *in vivo* studies that have shown disease protection used unmodified siRNA's that were not optimized for half-life [33], [34], similar to the data in our manuscript. An example of great interest and potential is the development of an siRNA drug candidate against RSV, where clinical trails demonstrated that low dosages of inhaled uncomplexed siRNA are a potent and easy way to administer antiviral therapeutics against respiratory viral diseases in humans (www.alnylam.com).

In the present study, we were also able to demonstrate the potential use of siRNA as a therapeutic alternative for the treatment of the animal respiratory viral disease caused by EHV-1 in a murine model. Besides showing the effectiveness of siRNA against EHV-1 when applied before infection, we demonstrated that siRNA application to mice after challenge infection was effective in reducing clinical symptoms and virus replication in the lungs of treated animals. This might be of particular importance in the case of EHV-1 outbreak situations where in contact or previously exposed horses are treated. Based on our results, it is conceivable that if siRNA's are applied during an outbreak, not only the severity of clinical signs of affected horses but also the number of affected horses and the magnitude of nasal shedding could be reduced, resulting in an overall reduction of the viral load in the population and improved control of the outbreak.

The effectiveness of the intranasal siRNA therapy needs to be evaluated in the target species before any firm conclusions can be drawn and experiments are under way to address this question. An important fact is the complex pathogenesis of EHV-1 in the horse compared to the murine model. Intranasal infection of mice usually remains restricted to the respiratory tissues, while in the horse EHV-1 targets the respiratory tract, the pregnant uterus and the central nervous system. However, by specifically targeting the nasal mucosa, the site of initial EHV-1 invasion and replication, this model of attenuation of disease in mice may be predictive for the horse.

The set of *in vitro* and *in vivo* experiments in the present study were designed to show that siRNA therapy targeting viral genes important for cell entry and replication is effective in decreasing severity of EHV-1 infection and disease. Not only was it possible to decrease the amount of infectious viral particles produced upon infection, it was also possible to decrease clinical signs. The findings have important implications in terms of treating and preventing outbreaks of EHV-1, as neither effective therapeutic nor prophylactic measures for this important disease are currently available. Taken together, the potential use of RNA interference in the control of EHV-1 infections by intranasal application of siRNA was demonstrated and could prove an effective means to control EHV-1 infections as metaphylactic and/or therapeutic measures in outbreak situations.

## Materials and Methods

### Cells, Viruses and siRNA's

Rabbit kidney (RK13) cells were maintained in minimum essential medium (MEM, Mediatech Inc.) supplemented with 10% fetal bovine serum (FBS), 100 U/mL penicillin and 0.1 mg/mL streptomycin (Mediatech Inc.), at 37°C under 5% CO_2_ atmosphere. Wild type EHV-1 strain Ab4 and the eGFP-expressing rAb4Δgp2 were propagated in RK13 cells [44]. The small interfering RNAs (siRNA's) against ORF33 (encoding gB) and ORF53 (encoding the helicase, Ori) were chemically synthesized (Ambion) based on the sequence of Ab4 (Genbank Sequence #AY665713) (Table 2).

**Table 2:** siRNA sequences.

name	gene target (protein)	siRNA sequence (5′→3′)^a^
sigB1	ORF33 (gB)	GGAUGGAGACUUUUACACCtt
		GGUGUAAAAGUCUCCAUCCtc
sigB2	ORF33 (gB)	GGAGAACGAGAUUUUCACGtt
		CGUGAAAAUCUCGUUCUCCtc
**sigB3**	**ORF33 (gB)**	**CGGAAAUCGAGGUUAUCAGtt**
		**CUGAUAACCUCGAUUUCCGtg**
siOri1	ORF53 (helicase)	CGAUAACCUCCUCAACAAUtt
		AUUGUUGAGGAGGUUAUCGtc
**siOri2**	**ORF53 (helicase)**	**CGAUGGUUCACCUCAACAAtt**
		**UUGUUGAGGUGAACCAUCGta**
siOri3	ORF53 (helicase)	CGGAGGUUUUUGAAAACGAtt
		UCGUUUUCAAAAACCUCCGtc
siLuc	Firefly Luciferase	Accession No: U47296
siGFP	eGFP	Accession No: U55761

a bold indicates siRNA's that showed significant reduction of EHV-1 replication.

### siRNA treatment and virus infection

Six-well plates of RK13 cells were treated with different concentrations of siRNA, ranging from 0 up to 75 pmol. The siRNA's were complexed with lipofectamine as per the manufacturers instructions (Invitrogen) and added to the cells in a total volume of 500 µL/well. After 2 hours of incubation at 37°C, 1.5 mL/well of growth media were added and incubated for 14 h. After washing, cells were inoculated with 100 plaque forming units (PFU)/well of eGFP-expressing rAb4Δgp2. One hour post infection (p.i.), medium was removed and infected cells were overlaid with fresh medium. Supernatants were collected at 24 h p.i. and cells were fixed with 10% formalin in phosphate buffered saline (PBS). To determine plaque sizes, at least 50 plaques per well were photographed and the average plaque areas were determined using the Image J software (http://rsb.info.nih.gov/ij). To determine extracellular viral titers, a standard plaque assay was used, essentially as described before [30]. Briefly, 10-fold dilutions of supernatants were plated on RK13 cells and 3 days p.i. cells were fixed with 10% formalin in PBS, stained with 0.3% crystal violet and plaques were counted.

To evaluate the effectiveness of siRNA administration in relation to time of infection, siRNA's were added 12, 6 and 2 h prior to EHV-1 infection; simultaneously with EHV-1 infection; and 1 h after EHV-1 infection.

### Western blotting

Western blot analyses were performed exactly as described previously [30]. To detect gB, mAb 3F6 was used at a 1∶500 dilution (kindly provided by Dr. G.P. Allen, University of Kentucky) [45]. Anti β-actin (Sigma), at a 1∶5000 dilution, was used as a control antibody. Anti-mouse IgG peroxidase conjugate was obtained from ImmunoResearch Laboratories and used at a dilution of 1∶5000.

### RNA extraction and real-time quantitative RT-PCR (qRT-PCR)

Six-well plates of RK13 cells were transfected with siRNA and infected 14 h later with wild-type Ab4 as described above. At 12 and 24 h p.i. cells were collected and following a freeze-thaw cycle, RNA was extracted using RNA STAT-60 (Tel-Test Inc. Friendswood, TX) essentially as described before [46]. Briefly, dried RNA pellets were dissolved in 100 µl RNase-free water and all RNA samples were DNase-treated with the Turbo DNA-free kit, according to the manufacturer's instructions (Ambion). cDNA was synthesized using the Thermoscript RT-PCR system (Invitrogen) according to the manufacturer's instructions using random hexamers with Thermoscript reverse transcriptase. qPCR was performed using the 7500-FAST real-time PCR system (Applied Biosystems) with reaction mixtures containing TaqMan Fast Universal PCR Master Mix, 900 nM primers, 250 nM probe and 5 µL cDNA, in a 20 µl total reaction volume. Parameters included 95 C for 20 sec to activate Taq polymerase, followed by 40 cycles of 95 C×3 sec and 60 C×30 sec. The primers and probes used are listed in Table 3 and the comparative C_T_ method for relative quantitation (2^−ΔΔCT^) was used with rabbit β-actin as the endogenous housekeeping gene [6], [44].

**Table 3:** Primers and Probes used in the study.

Real time PCR		
Gene	Primers (5′→3′) a	MGB probe
EHV-1 IR6	**F:** GCGAAGTACCCCTCGTTCATCT	TCGCGACACCGCCT
	**R:** ATGCTCGGGCGCTCCTACT	
EHV-1 gB	**F:** CGCTGAGGATGGAGACTTTTACA	CCACCGCCTACCGGATCCACC
	**R:** GGTGGTTCGATGCGTACG	
EHV-1 Ori	**F:** TGGTAACGGTGGGCCTTAGT	TTGATACGGCTCATT TCCACAGC
	**R:** GGGCTTGACGTAGGCAAACA	
rabbit β-actin	**F:** CGAGATCGTGCGGGACAT	AAGGAGAAGCTGTGCTACGTGGCGCT
	**R:** GCCATCTCCTGCTCGAAGTC	

Sequencing	
Gene	Primers (5′→3′) a
EHV-1 gB	**F:** TTTGGAAGCTGTGTTGTTAGAG
	**R:** TAATCACTGCGGTATTGTCCA
EHV-1 Ori	**F:** CACTTGCACCAGCCACGTTC
	**R:** CATGGGGGTAAAGATGGGCT

a **F:** Forward primer, **R:** Reverse primer.

### Animal experiments

All animal experiments were performed in accordance with the U.S. Animal Welfare Act, under the supervision of Cornell University's Animal Care and Use Committee, and were conducted as previously described with some modifications [30]. Three-to-four week-old female BALB/c mice (12 mice per group) were inoculated with varying concentrations and combinations of siRNA's and infected with 1×10^5^ PFU of the EHV-1 strain Ab4, as described in Table 1. Suspensions of siRNA's, with or without the transfection reagent lipofectamine in 20 µL OptiMEM or PBS respectively, and Ab4 in 20 µL MEM were administered intranasally (Table 1). Individual weights of mice were determined daily on the day of infection (day 0) up to day 14 p.i. Three mice from each group were euthanized to collect lungs on days 2 and 4 p.i. The left lobes were homogenized to determine viral titers on RK13 cells by standard plaque assays. The right lobes were fixed in 10% formaldehyde solution and processed for histopathological analysis. H&E-stained lung sections of three mice per group were scored in a double-blinded manner under the light microscope to determine the degree of inflammation, bronchiolar changes (hyperplasia and/or necrosis) and the presence of inclusion bodies. A value of 0 (normal), 1 (minimal), 2 (mild), 3 (moderate), 4 (marked) or 5 (severe) was assigned for each histological parameter and the scores per group were computed as the total lung score per group. In another set of experiments, five mice per group were euthanized on day 3 p.i. to collect lungs for viral titrations and additional determination of the viral genome load in these tissues by qPCR, which was performed exactly as previously described [44].

### Statistical analysis

Student's t-test for paired data was used to test for differences. Data given are the means and bars show standard deviations. Body weights were compared using non-parametric Wilcoxon-Whitney and Kruskal Wallis tests. All statistical calculations were performed with SAS vs. 9.1. (SAS Corporation, Cary, NC).
